# Characterization of Applicant Preference Signals, Invitations for Interviews, and Inclusion on Match Lists for Residency Positions in Urology

**DOI:** 10.1001/jamanetworkopen.2022.50974

**Published:** 2023-01-20

**Authors:** Ralph Grauer, Daniel Ranti, Kirsten Greene, Michael A. Gorin, Mani Menon, Saša Zorc

**Affiliations:** 1 Department of Urology, Icahn School of Medicine at Mount Sinai, New York, New York; 2 Department of Urology, University of Virginia, Charlottesville; 3 Department of Quantitative Analysis, University of Virginia Darden School of Business, Charlottesville

## Abstract

**Importance:**

Preference signals were to be implemented in over 15 specialties during the 2022-2023 residency match. Analyzing results from the implementation of signals during the American Urological Association (AUA) urology match may inform future behavior.

**Objective:**

To characterize applicant and program signal usage and results in the Society of Academic Urology and AUA databases with respect to interview invites and rank list creation.

**Design, Setting, and Participants:**

This cohort study involved all applicants and residencies in the 2021-2022 AUA match with data analysis conducted in April through July 2022.

**Exposures:**

Five signals indicating interest.

**Main Outcomes and Measures:**

Using verified match and survey data reported by applicants and programs, a logistic regression was performed on applicant factors associated with obtaining an interview—the main outcome (using inclusion on rank list as a proxy): age, gender, degree (MD or DO), dispersal of signal, US senior status, racial minority group status, Latino ethnicity, international medical graduate status, presence of a home program, AUA geographic section, and US Medical Licensing Examination Step 1 score. Applicant signal dispersal strategies were stratified by applicant and program competitiveness, as well as program behavior upon receipt of signal with respect to extending interviews and rank list ordering of applicants.

**Results:**

A total of 2659 signals were sent by 553 candidates (mean [SD] age, 27.4 [2.9] years; 179 female [32.4%], 243 racial minority candidates [61.2%]) submitting rank lists for 364 positions at 143 programs. Programs received a median (IQR) of 352 (295-411) applications and were signaled to a median of 16 (8-26) times each. In a logistic regression estimating interview status, geographic proximity (OR, 3.25; 95% CI, 2.05-5.15; *P* = .001) and signal status (OR, 6.04; 95% CI, 3.50-10.40; *P* < .001) were associated with receiving an interview. Using multiple imputation by chained equations to impute missing data and broadening the data set, male gender (OR, 0.64; 95% CI, 0.45-0.92; *P* = .04) and international medical graduate status (OR, 0.35; 95% CI, 0.15-0.81; *P* = .04) were negative variables, while MD degree (OR, 2.36; 95% CI, 1.27-4.36; *P* = .02) and US senior status (OR, 1.91; 95% CI, 1.13-3.23; *P* = .04) were positive variables.

**Conclusions and Relevance:**

This study of the usage and trends of the newly added preference signals reported the most common strategies for signal dispersal; in an analysis of factors involved in obtaining an interview, geographic similarity between applicant and program and preference signal usage were associated with successful applications.

## Key Points

**Question:**

How are preference signals used by applicants and programs participating in the residency match in urology?

**Findings:**

In this cohort study that included all 553 applicants and 143 residency programs in the urology match, preference signals were the most significant factor associated with an applicant receiving an interview but were not associated with applicants being ranked higher on programs’ rank order lists.

**Meaning:**

These results suggest that the introduction of preference signals was associated with applicant interviews but not applicant position on programs’ rank order lists.

## Teaser

This cohort study examines factors associated with US applicants receiving interviews for residency in urology programs following the implementation of preference signals at the matching stage.

## Introduction

The high opportunity cost of going unmatched has resulted in overapplication to residency programs during the match.^1^ In the 2021-2022 application cycle, urology programs in the US provided 364 available spots for approximately 600 registered applicants; ultimately, 553 applicants submitted a rank list, yielding a match rate of 66%.^2,3^ Applications to urology programs have risen over 40% in the last 2 years, with some programs receiving over 200 applications per vacancy. While the match algorithm efficiently pairs programs with interviewed candidates, a significant source of inefficiency in the job market for urology residents stems from limited interview slots: each department can only interview a small subsection of the applicants. Given this, programs face the difficult decision of which candidates to interview and struggle to uncover true, sincere interest from applicants among the many applications received.

In 2022, preference signals, also known as tokens, were implemented in the urology match to address the market inefficiencies of the historic system.^4^ Preference signals are a limited resource that conveys genuine interest in a binary manner. Urology applicants are given 5 tokens and are tasked with giving them to 5 separate programs to signal interest, in addition to applying to their full complement of programs. Thus, there is an inherent opportunity cost associated with sending each token. Applicants were instructed to not signal their home programs or any program where they performed a subinternship because genuine interest was assumed in these situations. Residency programs could use these tokens to inform their selection of candidates to interview but were advised not to use them to create their rank lists or for any other purpose.

Preference signaling mechanisms have been previously implemented in the 2021 otolaryngology match, the job market for economic PhDs, early decision college applications, and were slated to be widely incorporated in the 2023 match in over 15 specialties.^5,6,7^ The introduction of preference signaling has previously been shown to increase the likelihood of receiving an interview from 23% to 58% in the otolaryngology match.^7^ The efficiency of the preference signaling system is predicated on applicants strategizing where to send their tokens, as well as the departments’ understanding of the information the signal is conveying when making their interviewing decisions. Finding the optimal strategy for any single candidate is a mathematically intractable problem, but one where historical data and simulation can be very informative.^8^ Some resources have already emerged to help participants on both sides of this job market make informed decisions,^9^ and this is a topic we aimed to contribute to with our analysis. This study examined the most common strategies for token dispersal, the factors associated with obtaining an interview, and how signals affects programs’ ranking decisions.

## Methods

In this retrospective cohort study, anonymized applicant and program data were obtained directly from governing bodies of the urology match: the Society of Academic Urology (SAU) and the American Urological Association (AUA). Institutional review board approval and informed consent were not required due to the deidentified, non-Health Insurance Portability and Accountability Act–related nature of the data; in addition, the data were governed by the AUA and the SAU’s data-use agreements, to which all applicants agreed. This cohort study followed the Strengthening Reporting of Observational Studies in Epidemiology (STROBE) reporting guideline.

### Data and Study Population

The data were a combination of verified data from the match as well as survey data reported by applicants and programs. The survey data provided by the applicants included the following: anonymized identifying (ID) number, gender, home town and work geography (city, state, and country), degree (MD vs DO), age, self-reported race and ethnicity (African American and Black, Asian, Native American and Pacific Islander, White, other, and prefer not to say), self-reported Latino status, Alpha Omega Alpha (AOA) status, US Medical Licensing Exam (USLME) Step 1 and Step 2 scores, medical school geography, graduation date, whether or not their home institution has a urology training program, number of virtual interviews offered, and virtual interviews attended. Nonsurvey data provided by the AUA and the SAU on the applicants included to which residency program they matched and complete rank order list (ROL) information. The ROL information included residency programs each applicant signaled and the order of ranked residency programs. The program data included program geography (city and state), number of applications received, number of signals received, number of interviews held, number of signal interviews, matched applicant information (ID, home city, and home state), and complete ROL information.

In addition to provided data, several calculated metrics were generated. Competitiveness of a program was calculated by both the number of applications per interview and the applications per open spot. Geographic similarity was binarily assigned between applicant and program when an applicant had worked, studied, or lived in the same AUA Section (defined per AUA guidelines)^10^ as a program to which they were applying. Lastly, there were several binary categorical variables included, such as US senior status (ie, graduating in 2022), international medical graduate (IMG) status, and self-reported racial minority status (ie, African American and Black, Asian, Native American and Pacific Islander, and other). Using the data provided on each institution, residency programs were identified and rankings from Doximity rankings were assigned.

### Statistical Analysis

As the main outcome, we examined the association between applicant characteristics (including signal dispersal) and residency program interviewing by using logistic regressions with multiple imputations for missing data (eFigure in Supplement 1). We used a Little test to assess for true randomness in the missing data.^11^ First, we performed the analysis on a subset of applicants for whom no data were missing (182 applicants). We used applicant presence on programs’ ROL as a proxy for receiving an interview at that program. We used multiple imputations to impute the remainder of the full application list of each applicant. Variables for each round of imputation were all plausible data that could be used by a program to determine whether to give an applicant an interview. These variables included signal status, gender, racial minority status, degree (MD vs DO), US senior status, IMG status, whether the applicant’s home institution had a urology program, USMLE Step 1 score, and competitiveness of the program. The target variable was interview status, and the regression was performed on all individuals who were estimated to have applied to an institution. As previously described, 10 rounds of imputations were performed to achieve steady state estimations of the regression coefficients and statistical significance.^12^

Results of each round of imputations were aggregated and both coefficient and statistical significance were reported.^12^ In addition to the smaller cohort of 182 students, the same regression was performed on the entire cohort of 553 students. Multiple imputations for chained equations (MICE) were performed to impute the missing data for the entire cohort. Multiple imputation was used again to generate the application list as described above, and 10 rounds of regressions were performed and aggregated. Finally, odds ratios (OR) and 95% CIs were calculated using standard error values and the coefficient values. Correction for multiple hypotheses were not applied. The log-likelihood test was used to estimate significance of the calculated regression results. A sensitivity analysis was performed to validate the statistical effect of signal status on interview receipt using a stratified analysis examining for effect modifiers (eMethods and eFigure in Supplement 1).

Secondary analyses examined applicant signaling trends and program interviewing and ranking trends based on signal receipt. Signaling behavior was quantified on a per-applicant basis. Applicant competitiveness was calculated as the quartile of the number times each candidate appeared in the top 5 of any institution’s ROL. Statistical differences in signaling patterns sent to institutions were estimated via an analysis of variance test. In addition, applicant ROL information was compared with the complement of signaled programs, to evaluate whether signaled programs were ultimately ranked higher than non-signaled programs. Program characteristics were reported including signals received as a function of institutional Doximity ranking, percentage of applicants that signaled who received interviews, and programs’ ranking of applicants on ROL based on receipt of signal. Mann-Whitney U tests were used to compare ranked positions by signal status for both candidate and program rank lists. All analyses were performed in Python version 3.7.2 and R version 4.1.2 (R Project for Statistical Computing); the threshold for statistical significance was defined as a 2-sided *P* < .05.

## Results

During the 2021-2022 application cycle, 553 candidates submitted rank lists for 364 spots at 143 programs (mean [SD] age, 27.4 [2.9] years; 179 female [32.4%], 243 racial minority candidates [61.2%]) (Table 1). Applicants were overwhelmingly MD students (172 applicants [94.5%]). A total of 2659 signals were sent: 530 candidates (95.8%) sent the maximum of 5 signals, 4 candidates sent fewer than 5 but more than zero, and 19 candidates sent zero signals. While complete data on ROL and tokens were available, all other data were reported via survey responses. All data were missing at random, except for age, Latino status, racial minority status, and home program status, which were missing in high correlation to each other. Complete survey data was available for 182 candidates. For the remaining 371 candidates, survey data varied in terms of completeness.

**Table 1.** Baseline Applicant Characteristics in Both the Data Sets Used for Analyses

Characteristics	Survey responses, No. (%)^a^	
	No missing data (N = 182)	Entire match cohort (N = 553)^b^
Step 1 score, mean (SD)	243.0 (12.1)	240.8 (14.2)
Age, mean (SD), y	27.3 (2.7)	27.4 (2.9)
Gender		
Female	50 (27.5)	179 (32.4)
Male	132 (72.5)	371 (67.1)
Degree		
DO, MBBS	10 (5.5)	32 (8.1)
MD	172 (94.5)	365 (91.9)
Senior status		
Nonsenior	10 (5.5)	72 (13.3)
Senior	172 (94.5)	469 (86.7)
Racial minority candidate^c^		
Yes	105 (57.7)	243 (61.2)
No	77 (42.3)	154 (38.8)
Latino candidate		
Yes	168 (92.3)	358 (90.2)
No	14 (7.7)	39 (9.8)
IMG candidate		
Yes	180 (98.9)	526 (95.8)
No	2 (1.1)	23 (4.2)
Home program		
Yes	36 (19.8)	91 (22.9)
No	146 (80.2)	306 (77.1)
AUA section home		
Mid Atlantic	28 (15.4)	81 (15.8)
New England	8 (4.4)	29 (5.7)
New York	23 (12.6)	53 (10.4)
North Central	33 (18.1)	99 (19.3)
South Central	31 (17.0)	78 (15.2)
South Eastern	34 (18.7)	102 (19.9)
Western	25 (13.7)	70 (13.7)
AUA section school		
Mid Atlantic	30 (16.5)	78 (15.1)
New England	10 (5.5)	32 (6.2)
New York	24 (13.2)	62 (12.0)
North Central	35 (19.2)	107 (20.7)
South Central	32 (17.6)	74 (14.3)
South Eastern	33 (18.1)	101 (19.6)
Western	18 (9.9)	62 (12.0)

Abbreviations: AUA, American Urological Association; DO, doctor of osteopathic medicine; IMG, international medical graduate; MBBS, bachelor of medicine; MD, doctor of medicine.

^a^ Both data sets were used in 2 separate rounds of regressions, with multiple imputations used to generate application lists on a per-applicant basis.

^b^ Total missing responses: Step 1 score, 261 surveys; age, 156 surveys; gender, 3 surveys; degree, 156 surveys; senior status, 12 surveys; racial minority identity, 156 surveys; Latino ethnicity, 156 surveys; IMG status, 4 surveys; home program, 156 surveys; AUA section home, 41 surveys; AUA section school, 37 surveys.

^c^ Including participants identifying as African American and Black, Asian, Native American and Pacific Islander, and other.

Using the data set with complete data (182 applicants) in a logistic regression estimating interview status, we found that geographic proximity (based on mutual AUA status location of a program and a candidate’s school or home geography) (OR, 3.25; 95% CI, 2.05-5.15; *P* = .001), and signal status (OR, 6.04; 95% CI, 3.50-10.40; *P* < .001) were associated with receiving an interview. When using MICE to impute missing data and broadening the data set to all 553 applicants, male gender (OR, 0.64; 95% CI, 0.45-0.92; *P* = .04) and IMG status (OR, 0.35; 95% CI, 0.15-0.81; *P* = .04) were additional negative variables in receiving an interview, while MD degree status (OR, 2.36; 95% CI, 1.27-4.36; *P* = .02) and US senior status (OR, 1.91; 95% CI, 1.13-3.23; *P* = .04) were new positive variables in of receiving an interview (Table 2).

**Table 2.** Results of the Logistic Regression Estimating an Applicant’s Likelihood to Receive an Interview at a Given Program

Characteristic	No missing observations (n = 182)		Entire cohort (n = 553)	
	Odds ratio (95% CI)	*P* value	Odds ratio (95% CI)	*P* value
Male gender	0.68 (0.43-1.08)	.13	0.64 (0.45-0.92)	.04
MD degree	2.34 (1.06-5.17)	.06	2.36 (1.27-4.36)	.02
Work or school geography	3.25 (2.05-5.15)	.001	3.07 (2.15-4.39)	<.001
Signal status	6.04 (3.5-10.4)	.000	6.5 (4.27-9.9)	<.001
Racial minority identity	1.19 (0.76-1.86)	.47	1.27 (0.89-1.81)	.23
Latino ethnicity	1.25 (0.67-2.32)	.50	1.14 (0.69-1.87)	.63
IMG student	0.55 (0.17-1.81)	.35	0.35 (0.15-0.81)	.04
Home program	1.08 (0.62-1.87)	.80	1.05 (0.67-1.64)	.85
US senior	1.25 (0.59-2.66)	.57	1.91 (1.13-3.23)	.04
Applications per interview	1 (0.89-1.11)	.94	0.99 (0.91-1.09)	.91
Applications (100) per spot	0.93 (0.62-1.4)	.75	1.09 (0.79-1.51)	.60
Step 1 score	1.02 (0.93-1.11)	.68	1.02 (0.93-1.11)	.68

Abbreviations: IMG, international medical graduate; MD, doctor of medicine.

**Figure 1.**
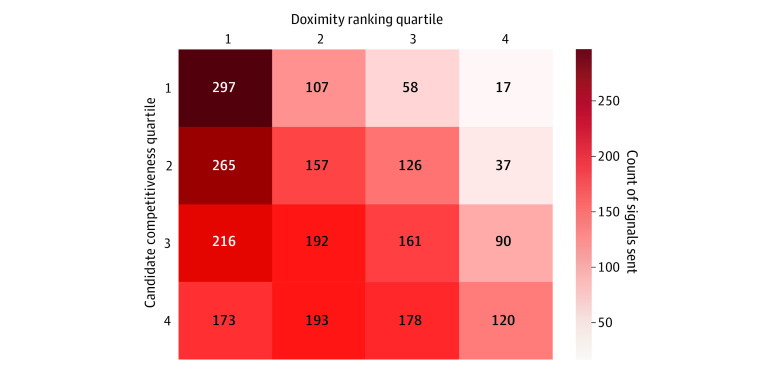
Heat Map of Applicant Signaling Trends Stratified by Applicant and Program Competitiveness Competitiveness defined as quartiles based on the number of times an applicant was ranked in the top 5 on any program’s rank order list and Doximity ranking quartiles, respectively.

**Figure 2.**
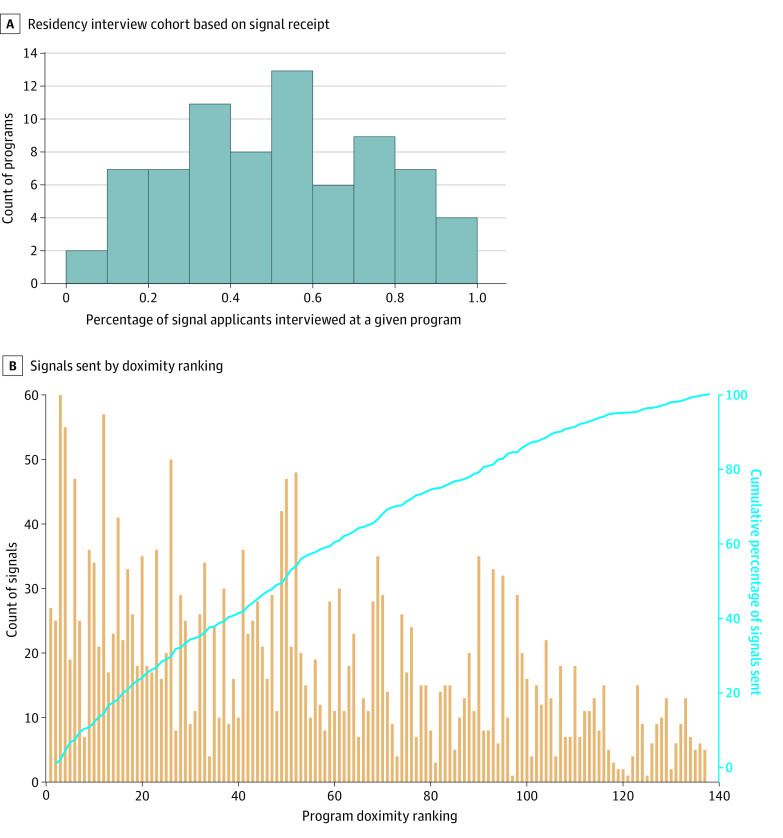
Histogram of Signaling Applicants and Signals Received by Residency Programs

Aggregated individual signaling patterns revealed the applicant dispersal strategies stratified by program competitiveness according to Doximity and candidate competitiveness metrics (Figure 1). Top-quartile candidates sent a statistically significantly higher proportion of their signals to top-quartile schools than bottom-quartile candidates, at 62.0% vs 26.1% (*P* < .001). Programs varied in the number of tokens they received and the proportion of signal applicants they chose to interview, with some interviewing only signaling applicants and some interviewing less than 10% (Figure 2). More specifically, programs received a median (IQR) of 352 (295-411) applications for a median of 42 (35-54) open interviews and were signaled to a median of 16 (8-26) times each. The median lowest ranked candidate to match on a program-by-program basis was ranked 9th (IQR, 6th-15th).

**Figure 3.**
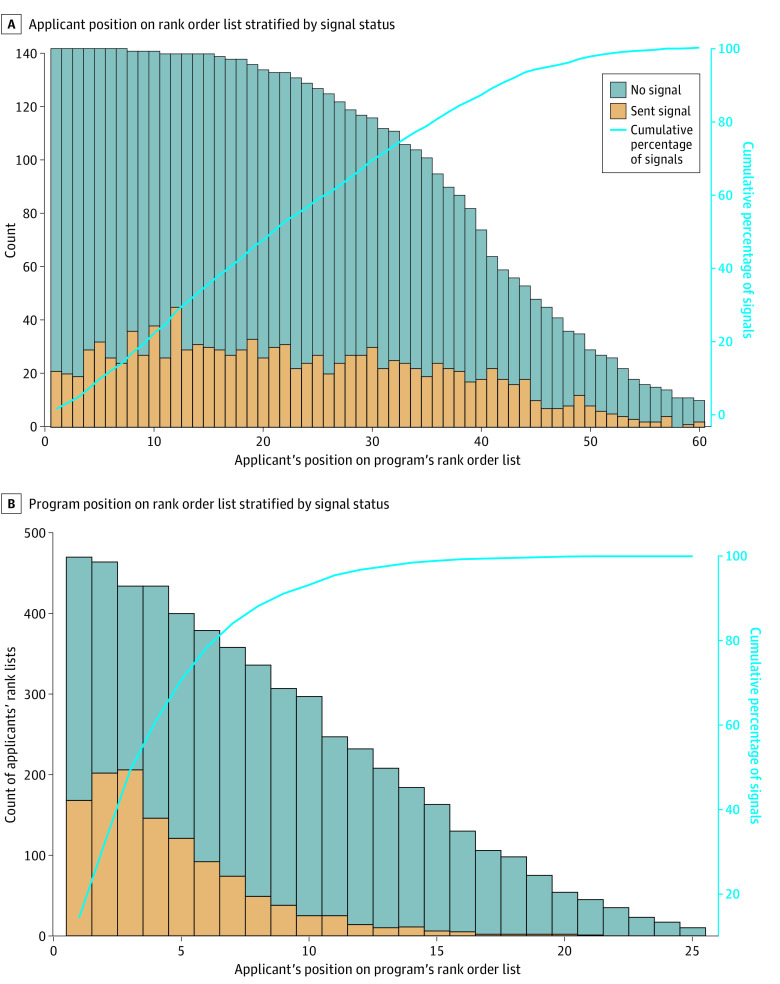
Rank Order List Stratified by Signal Status *P* values refer to Mann-Whitney U test used to compare ranked positions by signal status for both candidate and program rank lists. In panel A, more candidates were ranked in the top 5 from the nonsignal group when compared with the signal group (59 [10.7%] vs 40 [7.3%] of each group; *P* = .01); in panel B, programs that were signaled to were statistically significantly higher ranked than those that were not (median [IQR] rank: signaled, 4 [2-6] vs not signaled, 4 [4-13]; *P* < .001).

When looking at the geographical association between signals sent to institutions and the home or medical school region where an applicant worked, 117 applicants (21.9%) sent zero signals to institutions in the same geographical area as where they studied or lived, 109 (20.4%) sent 1 signal, 117 (21.9%) sent 2, 97 (18.2%) sent 3, 55 (10.3%) sent 4, and 39 (7.3%) sent 5. ROL lengths for institutions were a median of 3 fewer than their reported number of interviews. Overall, 117 (82.4%) programs ranked between zero and 9 fewer candidates than they reportedly interviewed; 8 (5.6%) ranked more candidates than they interviewed; 17 programs (12.0%) ranked 10 and fewer candidates. When comparing those who were ranked by a given institution, rankings for candidates who signaled to that institution were found to be significantly different than those who did not: more candidates were ranked in the top 5 from the nonsignal group when compared with the signal group (59 [10.7%] vs 40 [7.3%] of each group; *P* = .01) (Figure 3A). The median rank position for those who did signal vs those who did not signal, however, was identical at 21 (with signal: IQR, 12-33; did not signal: IQR, 10-32). When comparing program rankings on candidate's ROL, programs that were signaled to (median [IQR], 4 [2-6]) were statistically significantly higher ranked than those that were not (median [IQR], 4 [4-13]; *P* < .001) (Figure 3B).

## Discussion

In this cohort study, we used self-reported survey results as well as both verified ROL and signal preference match data to analyze applicant and program behavior following the new addition of preference signaling tokens to the 2022 urology match. We found heterogeneity in the way programs used preference signals to select applicants for interviews but on average the use of a signal increased applicants’ chances of getting an interview 6-fold compared with no signal being sent. Programs did not rank signaling applicants significantly higher than nonsignaled applicants. However, there was a higher proportion of nonsignaling applicants in the top 5 spots on programs’ ROL—perhaps because signaling to home and away subinternship programs was not allowed. An alternative explanation would be that an applicant had to be especially competitive to obtain an interview without a signal. When examining applicant token dispersal stratified by applicant and program competitiveness, we found the applicant competitiveness was associated with the complement of programs to which they sent their tokens. Only about 70% of an applicant’s signaled programs ultimately appeared in a top 5 positions on the ROL, indicating a preference shift. These findings suggest that preference signals helped applicants obtain interviews at signaled programs, but the interviews themselves determine ultimate order for both program and applicant final preference lists. These findings are consistent with the instructions given to applicants and programs by the SAU and AUA for the intended use of preference signals.

Urology is the second specialty to adopt preference signals to decrease pre-interview application congestion by attempting to uncover genuine applicant interest. With the upcoming 2022-2023 match, over 15 medical and surgical specialties will implement some form preference signaling, most of which will offer 3 to 8 signals of equal value. Notable exceptions include obstetrics and gynecology, which will offer 18 signals (3 tier 1 and 15 tier 2), and orthopedic surgery, which will offer 30 tokens of equal value. With the increase in the number of signals, the opportunity cost of an individual signal may paradoxically increase as they begin to function as de facto interview caps—a consequence outside the original intent. Forged to alleviate application congestion and allow residency programs to select genuinely interested candidates to interview, preference signals will be widely adopted in the upcoming match.

Preference signals are not a novel innovation, and have been well described in existing economics and medical research.^8,13,14^ In fact, several innovations have been explored for assuaging the burden of excessive interviewing in medical matching markets. Interview caps have been argued for, but there is concern for potential antitrust litigation because of applicants going unmatched.^15^ With the goal being to create less, but more fruitful interviews, Wapnir et al^8^ proposed and modeled an interview match wherein candidates and programs privately submit their preferences for interviews. The subsequent invites would be decided by the same National Resident Matching Program (NRMP) matching algorithm as the final match, which would be performed following the round of interviews. A key point in their analysis is that matched interviews may be more likely to convert into a match when compared with the implementation of simple interview caps, as programs are able to convey more precise preferences prior to interviews. In a similar attempt to decongest the market, an early result acceptance program (ERAP) has been proposed and a stakeholder analysis performed on matched residency applicants and OBGYN program directors.^16^ Based on the premise that 77% of surveyed applicants received at least 1 interview in their top 3 residency programs, ERAP applicants would receive 3 applications. Modeling this, they concluded that if 15% to 30% of all positions are offered and filled in the ERAP (prior to the final match) there would be approximately 2000 to 4000 less interviews conducted overall while saving $1 to $2 million in lost faculty time. At the same time, ERAP-matched applicants would save interviewing costs and stress, while those not obtaining a position would continue with the regular match.

The NRMP reported that there were over 36 000 postgraduate year 1 residency positions across all specialties offered in 2022.^17^ With over 60 applications sent per applicant and dozens of interviews per vacancy, there is clearly significant inefficiency in the interview methodology. Ultimately, signaling tokens are merely 1 tool in the approach to streamline the application process and optimize the matching market—a feat that will likely require a multidimensional approach.

### Strengths and Limitations

This study has notable strengths. To our knowledge, this is the first study to use verified match ROL data to analyze and report factors associated with interview receipt, particularly following the newly implemented preference signals.

Our study also had several limitations. Despite having verified ROL data, the applicant and program data were based on optional survey data submitted concurrently with the rank list; thus, all applicants and programs completed some part of the survey, but complete data was available for 182 of 553 applicants. Step 1 score, in particular, was not available in 261 of 553 total candidates (47.2%); however, the ongoing utility of Step 1 as a matching tool is ambiguous given the shift to a pass-fail format. Additionally, our main outcome (inclusion of a program on an applicant’s rank order list) was a proxy for receiving an interview, not a true measure of interview invitation. Naturally there are applicants, albeit likely few, that receive an interview and choose not to attend or attend and then choose to not rank that program; these events could not be captured and represent a limitation of our analysis. We also did not have match data from prior years, which would have allowed for a more robust analysis of the signal implementation on a broad scale. Also, Doximity reputation rankings were used in the analysis as the basis for competitiveness. Although these rankings are easily available, they are subjective, imperfect, and self-perpetuating. Moreover, urology is a specialty with relatively few training spots and shorter rank order lists and thus signals usage may differ in other larger specialties.

## Conclusions

In this cohort study of the usage and trends of the newly added preference signals, we used verified ROL, signal preference from SAU and AUA match data, and self-reported survey data to analyze applicants and programs. In the cohort with complete data, we found geographic concordance between applicant and program, as well as signal usage to be associated with interview receipt. Using MICE to impute missing data, we additionally found female gender, MD degree (compared with DO), US senior status (compared with reapplicant), and US medical student (compared to IMG) to be factors positively associated with receiving an interview.

The association between sending a signal to an institution and obtaining an interview suggests that the new preference signals successfully perform their function. Because of this, fields other than urology may benefit from their introduction. Several questions of practical relevance remain open for future studies: what the ideal number of tokens is to provide candidates, what the optimal token dispersal strategy is, and whether alternative mechanisms (eg, a centralized interview match algorithm) could improve interview decisions further. Answering those questions may require a different methodological approach, such as game theory–based simulations.

## Notes

Supplement 1.
eMethods.

eResults.
**eFigure.** Flow chart

Supplement 2.
Data Sharing Statement
