# Error in Table, Abstract, and Results

**DOI:** 10.1001/jamanetworkopen.2023.3305

**Published:** 2023-02-27

In the Original Investigation titled “Characterization of Applicant Preference Signals, Invitations for Interviews, and Inclusion on Match Lists for Residency Positions in Urology,”^1^ published January 20, 2023, the row labels in Table 1 for racial minority candidate, Latino candidate, IMG candidate, and home program were transposed—“No” should have appeared above “Yes.” Data from this table appeared in the Abstract and Results. Racial minority candidates should have appeared as 154 candidates (38.8%), and applicants that were MD students as 365 candidates (91.9%). This article has been corrected.^1^
