# Supplementary material for: Characterization of Applicant Preference Signals, Invitations for Interviews, and Inclusion on Match Lists for Residency Positions in Urology
Source: JAMA Netw Open. 2023 Jan 20;6(1):e2250974. doi: 10.1001/jamanetworkopen.2022.50974 (PMC9860522; doi:10.1001/jamanetworkopen.2022.50974)
Supplement: Supplement 1. — eMethods. eResults. eFigure. Flow chart [file jamanetwopen-e2250974-s001.pdf]

Supplemental Online Content

Grauer R, Ranti D, Greene K, Gorin MA, Menon M, Zorc S. Characterization of applicant preference signals, invitations for interviews, and inclusion on match lists for residency positions in urology. *JAMA Netw Open.* 2023;6(1):e2250974. doi:10.1001/jamanetworkopen.2022.50974

eMethods.

eResults.

eFigure 1. Flow chart

This supplemental material has been provided by the authors to give readers additional information about their work.

© 2023 Grauer R et al. *JAMA Network Open.*

eMethods.

Several sensitivity analyses were performed to validate the statistical effect of signal status on receipt of an interview. All findings were evaluated with the complete dataset, as it was the most reliable dataset at the disposal of the authors. Effect modifiers occur when the magnitude of the effect of the primary exposure of the outcome of interest (in this case predicted association between signal status and interview status) vary along some third variable. To test the presence of effect modifiers, we performed a stratified analysis along the work/school geography Boolean to partition the data. In addition, we added in interaction terms between signal and geographical term, and signal and IMG status. Signal status p value in the resulting aggregated regression was observed after the addition of these terms.

© 2023 Grauer R et al. *JAMA Network Open.*

eResults. For the stratified analysis, candidates with geographical ties ($p = 0.007$) and without geographical ties saw signal retain statistical significance ($p < 0.001$). For candidates without geographical ties, the odds ratio of signal status was 9.3; for candidates with a geographical tie, the odds ratio was 3.2. In both cases, the effect persisted in a statistically significant manner across the stratification. In the model with interaction terms, signal status remained statistically significant ($p < 0.001$; odds ratio 9.3).

© 2023 Grauer R et al. *JAMA Network Open.*

eFigure. Flow chart

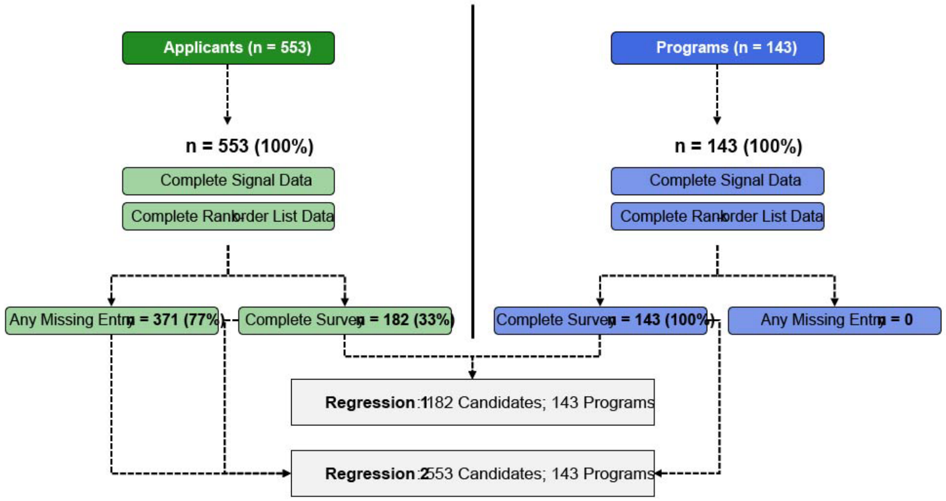

© 2023 Grauer R et al. *JAMA Network Open.*
