# Supplementary material for: Characterization of Applicant Preference Signals, Invitations for Interviews, and Inclusion on Match Lists for Residency Positions in Urology
Source: JAMA Netw Open. 2023 Jan 20;6(1):e2250974. doi: 10.1001/jamanetworkopen.2022.50974 (PMC9860522; doi:10.1001/jamanetworkopen.2022.50974)
Supplement: Supplement 2. — Data Sharing Statement [file jamanetwopen-e2250974-s002.pdf]

Data Sharing Statement

Grauer. Characterization of Applicant Preference Signals, Invitations for Interviews, and Inclusion on Match Lists for Residency Positions in Urology. *JAMA Netw Open*. Published January 20, 2023. doi:10.1001/jamanetworkopen.2022.50974

Data
Data available: No
